# Fruits of their labour: biotransformation reactions of yeasts during brewery fermentation

**DOI:** 10.1007/s00253-022-12068-w

**Published:** 2022-07-19

**Authors:** Natalia Svedlund, Simon Evering, Brian Gibson, Kristoffer Krogerus

**Affiliations:** 1 Chair of Brewing and Beverage Technology, Institute of Food Technology and Food Chemistry, Technische Universität Berlin, Ackerstraße 76, 13355 Berlin, Germany; 2 VTT Technical Research Centre of Finland, Tietotie 2, P.O. Box 1000, FI-02044 VTT Espoo, Finland

**Keywords:** Polyfunctional thiol, Terpene, β-lyase, β-glucosidase, Yeast, Fermentation, Beverages, Biotransformation

## Abstract

**Abstract:**

There is a growing appreciation for the role that yeast play in biotransformation of flavour compounds during beverage fermentations. This is particularly the case for brewing due to the continued popularity of aromatic beers produced via the dry-hopping process. Here, we review the current literature pertaining to biotransformation reactions mediated by fermentative yeasts. These reactions are diverse and include the liberation of thiols from cysteine or glutathione-bound adducts, as well as the release of glycosidically bound terpene alcohols. These changes serve generally to increase the fruit and floral aromas in beverages. This is particularly the case for the thiol compounds released via yeast β-lyase activity due to their low flavour thresholds. The role of yeast β-glucosidases in increasing terpene alcohols is less clear, at least with respect to fermentation of brewer’s wort. Yeast acetyl transferase and acetate esterase also have an impact on the quality and perceptibility of flavour compounds. Isomerization and reduction reactions, e.g. the conversion of geraniol (rose) to β-citronellol (citrus), also have potential to alter significantly flavour profiles. A greater understanding of biotransformation reactions is expected to not only facilitate greater control of beverage flavour profiles, but also to allow for more efficient exploitation of raw materials and thereby greater process sustainability.

**Key points:**

• *Yeast can alter and boost grape- and hop-derived flavour compounds in wine and beer*

• *β-lyase activity can release fruit-flavoured thiols with low flavour thresholds*

• *Floral and citrus-flavoured terpene alcohols can be released or interconverted*

## Introduction

The use of hops in the brewing process has increased dramatically in the past decades as a result of greater consumer demand for heavily hopped India Pale Ale (IPA)-style beers (Lafontaine and Shellhammer 2019a). In the USA, average hop use in breweries increased almost 50% from 2011 to 2019 (Lafontaine and Shellhammer 2019a; Cantwell and Swersey 2019). At the same time, global hop production has also grown. While hops were traditionally used mainly to impart bitterness and noble hop aroma (i.e. floral, herbaceous and woody) to beer, hops are today often used to impart a more intense hop aroma and taste, and newly developed hop varieties may contribute a wide range of fruity flavours to beer. In addition to increasing hop usage and diversity of hop varieties, brewers also exploit the ability of yeast to produce a wide range of flavour compounds in their attempts to meet consumer demand for more flavourful and diverse beers (Holt et al. 2019). A number of such flavour compounds are formed by enzymatic action from precursors originating from hops and malt. The term ‘biotransformation’ is often used to describe these types of flavour-releasing reactions during brewery fermentations. Exploiting these reactions is not unique to beer, as they occur and are exploited during wine and other beverage fermentations as well. In fact, the research on biotransformation reactions occurring during winemaking pre-dates that in brewing by many years, and the former has inspired and informed the latter. In this review, we focus on the recent work related to brewing yeast biotransformations but also refer to relevant work from the winemaking sphere.

Biotransformation reactions are of interest to brewers and other beverage producers for numerous reasons. Foremost, they allow for an increased yield of aroma-active compounds in the final product. As will be discussed in more detail below, many of these compounds are present in conjugated odourless forms in the raw materials, often at concentrations many-fold higher than that of the free form. Through biotransformation, increased concentrations of the free aroma-active forms can be obtained in the beverage, in addition to compounds not originally found in the raw materials. This allows the introduction of novel flavours to the beverage by converting precursors from the raw materials. As hops typically constitute the bulk of the raw material costs in brewing and they exert a considerable agricultural demand (Denby et al. 2018; Hauser and Shellhammer 2019; Lafontaine and Shellhammer 2019b), there is also potential to increase the sustainability and lower the environmental impact of the brewing process by exploiting biotransformation to produce similar flavour from less raw material.

**Fig. 1:**
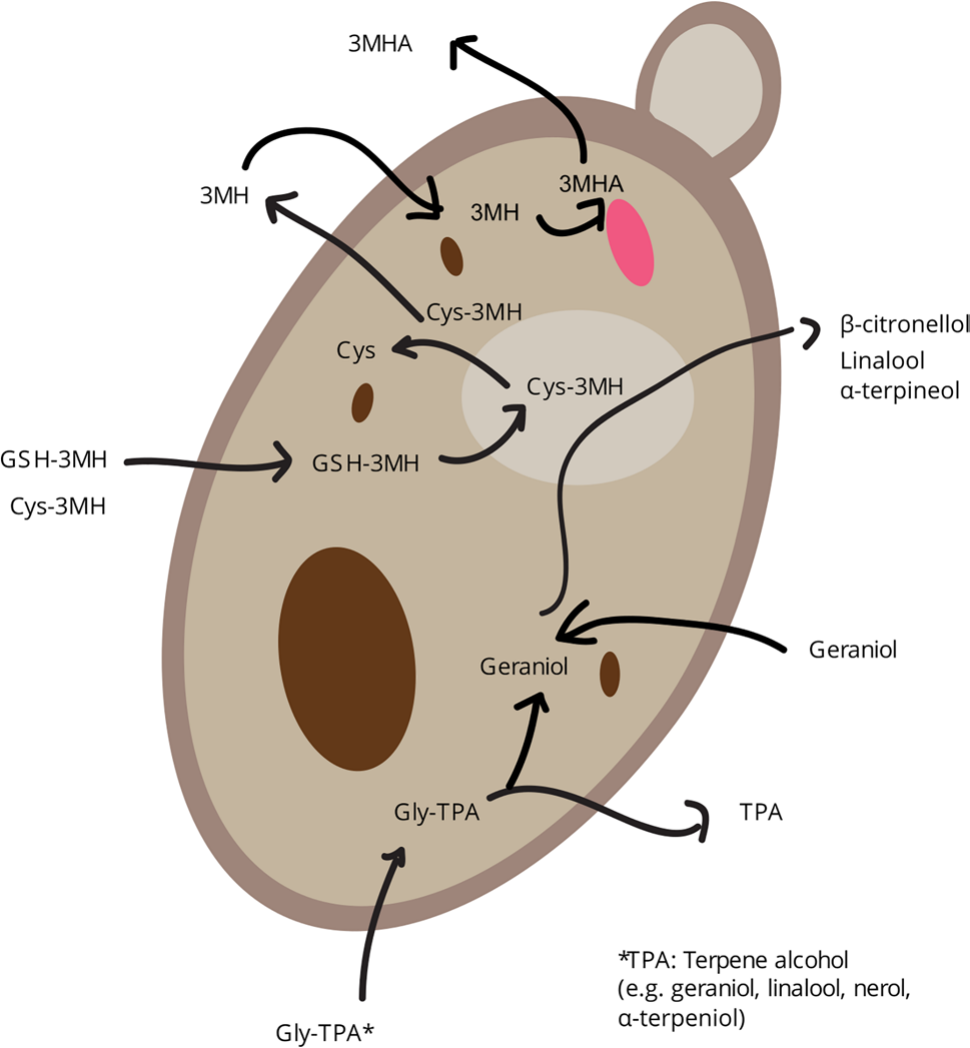
An overview of the biotransformation reactions occurring in yeast. Abbreviations: 3MH 3-mercaptohexanol, 3MHA 3-mercaptohexyl acetate, Cys cysteine, GSH glutathione, TPA terpene alcohol. Credit: Henrik Svedlund

In this mini-review, we cover the latest developments in using biotransformation as a tool to release additional flavour during beverage fermentations. The main focus is on beer fermentation and precursors derived from hops, but other beverage fermentations are covered as well. We discuss four different types of reactions, including β-lyase activity to release polyfunctional thiols, β-glucosidase activity to release terpene alcohols, reduction of terpene alcohols and esterification of polyfunctional thiols and terpene alcohols (Fig. 1). Furthermore, we also present strategies that can be used to enhance biotransformation capabilities of yeast strains and process changes that increase the yield of aroma-active compounds.

## Polyfunctional thiols

Polyfunctional, sulphur-containing, thiols are an important group of compounds that influence the aroma of a broad range of beverages, both fermented and non-fermented (Roland et al. 2011; Holt et al. 2019; Bonnaffoux et al. 2021). These molecules typically have strong aroma and are detectable at very low concentrations (ng per litre levels). The aromas of such thiols can range from unpleasant (e.g. onion- and skunk-like) to pleasant (e.g. black currant and tropical fruits) (Table 1). In fermented beverages, such as wine, beer and saké, two pleasant-smelling volatile thiols are of particular importance: 3-mercaptohexan-1-ol (3MH; also referred to as 3-sulfanylhexan-1-ol, 3SH) and 4-mercapto-4-methylpentan-2-one (4MMP; also referred to as 4-methyl-4-sulfanylpentan-2-one, 4MSP) (Darriet et al. 1995; Tominaga et al. 1998a; Kishimoto et al. 2006; Kishimoto et al. 2008a; Iizuka-Furukawa et al. 2017). Wines made from Sauvignon blanc grapes, for example, have been shown to contain particularly high concentrations of these thiols (Roland et al. 2011), while recent studies have shown they are vital to fruity hop aroma in modern highly hopped beer (Kankolongo Cibaka et al. 2017; Dennenlöhr et al. 2020; Biendl et al. 2021). In addition to the two previously mentioned thiols, 3-sulfanyl-4-methylpentan-1-ol (3S4MP) and 3MP (3-mercaptopentanol; also referred to as 3-sulfanylpentan-1-ol, 3SP) have also been to shown to contribute to the fruity aroma of certain hop varieties, such as Nelson Sauvin (Sarrazin et al. 2007; Takoi et al. 2009a).

**Table 1:**
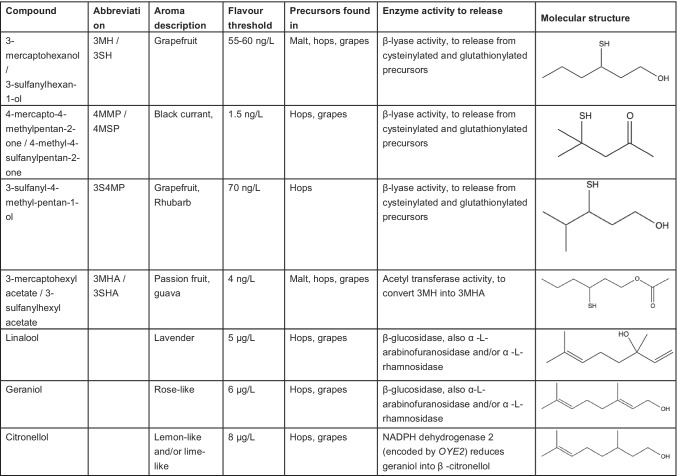
A summary of hop- and grape-derived flavour compounds that can be released or formed through biotransformation reactions

(Kishimoto et al. 2006; Czerny et al. 2008; Takoi et al. 2009a, 2009b, 2010a; Lafontaine et al. 2021)

### Thiol precursors in raw materials

While hops and grapes contain volatile thiols that can be directly transferred to the fermented beverage (Kishimoto et al. 2008a; Takoi et al. 2009a; Capone et al. 2011, 2012), the majority of volatile thiols that are present in beer, wine and saké are released by enzymatic action from glutathionylated or cysteinylated precursors found in the malt, hops, grapes and rice (Tominaga et al. 1998b; Peyrot des Gachons et al. 2000; Kishimoto et al. 2008b; Roland et al. 2010, 2016; Gros et al. 2012; Iizuka-Furukawa et al. 2017; Chenot et al. 2019). Early work using bacterial enzymes revealed that free volatile thiols could be released from the conjugated precursors by action of a carbon–sulphur lyase enzyme (Tominaga et al. 1998b; Wakabayashi et al. 2004). In *Saccharomyces cerevisiae*, the main β-lyase enzyme involved in releasing volatile thiols from conjugated precursors is encoded by the *IRC7* gene (Howell et al. 2005; Thibon et al. 2008; Roncoroni et al. 2011). In addition to *IRC7*, the β-lyase enzyme encoded by the *STR3* gene has also been shown to release volatile thiols from cysteinylated 3MH and 4MMP (Holt et al. 2011). The β-lyase activity of yeasts will be discussed in more detail in the following section.

The amount of thiol precursor found in the raw materials varies greatly with variety. In grapes, where barely any free thiols are detectable, high 3MH and 4MMP precursor concentrations have been found in, e.g. Sauvignon blanc, Gewürztraminer, Semillon, Chardonnay and Riesling grapes (Concejero et al. 2014; Bonnaffoux et al. 2017). Precursor concentrations in these varieties have even been measured in the mg/L range. Hops, which also contain considerable amounts of free thiols, have also been shown to contain high concentrations of 3MH and 4MMP precursors (Roland et al. 2016). Concentrations of 6.5 mg/kg 3MH precursors have been reported for Cascade hops, and these concentrations are 1000-fold higher than the concentrations of free 3MH. In addition, a recent survey revealed the presence of cysteinylated and glutathionylated sulphanylalkyl aldehydes and acetates in hops and grapes for the first time (Chenot et al. 2022a). Despite a large pool of thiol precursors existing in the fermentation media, only a small fraction is typically converted into the free form during fermentation. In wine, the fractions of glutathionylated or cysteinylated precursors in the grape must that are converted into free thiols typically remain below 1% regardless of yeast strain or grape variety (Murat et al. 2001; Subileau et al. 2008b; Roland et al. 2011; Bonnaffoux et al. 2018). A similarly low conversion yield (0.1–0.5%) has been observed in beer wort (Nizet et al. 2013; Michel et al. 2019; Chenot et al. 2019, 2021). This low conversion could be linked to poor activity of β-lyase activity in acidic media and inhibition by polyphenols (Chenot et al. 2022b).

From a process point-of-view, the temperature during beer fermentation was recently shown to affect the release of various thiols from their conjugated precursors (Chenot et al. 2021). However, the effect of temperature varied between thiols, with 3MH and 3MP (3-mercaptopentanol; also referred to as 3-sulfanylpentan-1-ol, 3SP) release highest at 18–24 °C and 3S4MP (3-sulfanyl-4-methylpentan-1-ol) release highest at 28 °C. A post-fermentation maturation at 4 °C for up to 5 days was shown to steadily increase thiol concentrations (Chenot et al. 2021). In addition to temperature, addition of exogenous enzymes to beer (e.g. cystathionine β-lyase and apotryptophanase) was recently shown to release thiols from cysteinylated precursors (Chenot et al. 2022b). However, conversion rates were on a similar level to those observed by brewing yeast. Further addition of a γ-glutamyl transpeptidase enzyme also allowed the release of thiols from glutathionylated precursors (Chenot et al. 2022b). Preliminary unpublished work also suggests that adding hops to the mash could increase the amount of volatile thiols released into the beer during fermentation (Burns 2021). This might be a result of protease activity in the mash, which could release or modify the conjugated precursors in the hops. Finally, using copper in the brewing or winemaking process, or when treating raw materials (e.g. hops or grapes), has been shown to decrease 4MMP content of the beverage (Swiegers and Pretorius 2007; Kishimoto et al. 2008b; Morimoto et al. 2010).

### Thiol release through β-lyase activity

The release of volatile thiols from conjugated precursors during yeast fermentation has been directly linked to *IRC7* expression (Thibon et al. 2008). The resulting β-lyase enzyme is involved in sulphur metabolism and amino acid biosynthesis, and expression of the gene is linked to the nitrogen catabolite repression process (Subileau et al. 2008a; Thibon et al. 2008)*.* In the presence of favourable nitrogen sources, such as ammonium, *IRC7* is repressed. *IRC7* regulation has been linked to the transcriptional activator Gln3p and its inhibitor Ure2p. Deletion of the activator-encoding *GLN3* decreased thiol release, while deletion of the inhibitor-encoding *URE2* increased thiol release (Thibon et al. 2008). Strains carrying loss-of-function mutations in *URE2* have also been shown to release higher amounts of thiols (Dufour et al. 2013). Selection of *ure2* mutants has been successfully demonstrated through growth on agar plates containing the toxic ammonium analogue methylamine and proline as sole nitrogen sources (Salmon and Barre 1998). A number of brewing strains carrying heterozygous nonsense mutations in *URE2* have also been identified among the ‘United Kingdom’ sub-group of the ‘Ale beer’/’Beer 1’ clade (Krogerus et al. 2021). Expression of *IRC7* in *S. cerevisiae* is also affected by its location in the sub-telomeric region of the right arm of chromosome VI (Holt et al. 2019). This region is silenced by the *SIR2*-encoded histone deacetylase, and both *SIR2* deletion and mutations in the subunit-encoding *SIR3* and *SIR4* genes have shown to increase *IRC7* expression (Ehrentraut et al. 2010; Samel et al. 2017).

In addition to regulation, thiol release by Irc7p is also affected by polymorphisms in the coding sequence of *IRC7.* The most well reported of these is a 38-bp deletion (*IRC7*^*S*^), which results in the formation of a truncated 360 amino acid protein with considerably lower activity than the full-length 400 amino acid Irc7p (Roncoroni et al. 2011). This 38-bp deletion is surprisingly widespread among wine strains and found in heterozygous form among many ‘Mosaic beer’/ ‘Beer 2’ brewing strains, but it is not so common in ‘Ale beer’/’Beer 1’ brewing or other domesticated strains (Cordente et al. 2019; Ruiz et al. 2021; Krogerus et al. 2021). The *S. cerevisiae* S288C reference genome also contains the 38-bp deletion in *IRC7*. Strains carrying either homozygous or heterozygous alleles of *IRC7*^*S*^ release lower amounts of thiols during fermentation (Roncoroni et al. 2011; Belda et al. 2016; Cordente et al. 2019). Along with *IRC7*^*S*^*,* a number of other inactivating mutations in *IRC7* have been identified. These include the T185A mutation, which reduces enzyme activity and thiol release by around 50% (Cordente et al. 2019). When coupled with other mutations, like K43R, P146R, G253R, G321D and E323G, enzyme activity and thiol release are decreased further. The T185A mutation is common among both wine and brewing strains (Cordente et al. 2019; Krogerus et al. 2021).

As β-lyase activity occurs inside the cell, the glutathionylated or cysteinylated precursors need to be transported inside the cell for any thiol release to occur. Limited studies on the topic have identified the *OPT1*-encoded oligopeptide transporter as the main transporter for glutathionylated 3MH and 4MMP (Subileau et al. 2008b; Santiago and Gardner 2015; Cordente et al. 2015). Deletion of *OPT1* significantly reduces release of both 3MH and 4MMP from their glutathionylated precursors, and 3MH release in particular is affected. Furthermore, deletion of *ECM38*, encoding a vacuolar transpeptidase, also increases release of 3MH from Glu-3MH (Cordente et al. 2015). Transport of cysteinylated precursors is not as well established. Preliminary work by Subileau et al. (2008a) suggested the *GAP1*-encoded general amino acid permease was involved, but no change in thiol release from cysteinylated precursors was observed when nine genes encoding known cysteine-transporting permeases, including *GAP1*, were deleted (Santiago and Gardner 2015).

A number of strategies have successfully been used to improve thiol release in yeast strains. Overexpression of the native full-length *IRC7* and *STR3* in wine strains has been shown to increase the release of 3MH and 4MMP (Holt et al. 2011; Roncoroni et al. 2011). Overexpression of the *tnaA*-encoded tryptophanase from *Escherichia coli* in wine strains also released 10- to 95-fold higher concentrations of 3MH in wine (Holt et al. 2011; Kiene et al. 2021). As described above, deletion of *URE2* also increased 3MH and 4MMP concentrations fourfold in synthetic juice media (Thibon et al. 2008).

Breeding or hybridization has also been used to construct yeast strains with enhanced β-lyase activities. *ure2* mutations were introduced to various wine strains by first crossing them with a lab strain carrying a non-functional *URE2* allele and then backcrossing them with the wine strains (Dufour et al. 2013). *ure2* hybrids produced higher levels of both 3MH and 4MMP during wine fermentations. In a recent study, brewing yeasts with CRISPR/Cas9-aided mating type switching were bred in an attempt to construct strains with enhanced thiol release abilities (Krogerus et al. 2021). Using *IRC7* sequences as a marker, crosses between different brewing strains and between brewing and wild strains were performed. Selected hybrids produced higher amounts of 4MMP and 3MH-acetate. Interspecific hybridization between *S. cerevisiae* and *Saccharomyces uvarum* strains has also been used to enhance thiol release during both wine (Masneuf-Pomarède et al. 2002; da Silva et al. 2015) and beer fermentations (Krogerus et al. 2022). In the latter study, enhanced concentrations of 4MMP, 3MH and 3MH-acetate in beer were obtained through fermentation with brewing strains crossed with selected *S. uvarum* strains. Indeed, from limited studies, it appears as if *S. uvarum* strains tend to have higher thiol release abilities than *S. cerevisiae* strains (da Silva et al. 2015; Knight et al. 2018). A possible explanation could be that *IRC7* is located further away from the telomeres in *S. uvarum* and unlikely to be affected by sub-telomeric silencing (Holt et al. 2019). A recent study in wine also revealed high thiol release by a strain of *Saccharomyces kudriavzevii*, highlighting the potential of other *Saccharomyces* species as well (Pérez et al. 2022).

Considerable β-lyase activity has been observed in non-*Saccharomyces* yeast as well. In wine fermentations, *Metschnikowia pulcherrima*, *Torulaspora delbrueckii*, *Lachancea thermotolerans* and *Candida zemplinina* have been shown to release high levels of 3MH or 4MMP in comparison to *S. cerevisiae* wine strains (Anfang et al. 2009; Zott et al. 2011; Belda et al. 2016). To overcome the often poor fermentation performance of such non-*Saccharomyces* strains, co- or sequential fermentation with a *Saccharomyces* yeast can be employed. Studies focusing on the β-lyase activity of non-*Saccharomyces* yeasts during beer fermentations are however limited. In a recent study, the ability of two *T. delbrueckii* strains to release 3MH in beer was shown to be similar to the included *S. cerevisiae* and *Saccharomyces pastorianus* strains (Michel et al. 2019). β-lyase activity and 3MH release have also been demonstrated in *Lactobacillus plantarum* during grape juice fermentations, highlighting that the ability is not limited to yeast (Takase et al. 2018).

Yeast can be screened for β-lyase activity using growth media containing a cysteinylated substrate as the sole nitrogen source. Growth on S-methyl-L-cysteine (SMC) as the sole nitrogen source has been shown to positively correlate with β-lyase activity (Belda et al. 2016). However, when recently applied to brewing yeast, growth on SMC could not discriminate strains with good thiol release very well (Michel et al. 2019; Krogerus et al. 2021). High β-lyase activity has also been associated with the ability to grow on cysteine as the sole nitrogen source (Santiago and Gardner 2015). This was recently exploited to select brewing yeasts with enhanced β-lyase activity (Krogerus et al. 2021).

### Acetylation of 3-mercaptohexanol

As described above, yeasts play a significant role in liberating free polyfunctional thiols from their cysteinylated or glutathionylated forms during fermentation. Biotransformation of polyfunctional thiols by yeast is not however limited to those reactions involving β-lyase. Fermented beverages, such as wine and beer, are also known to contain acetylated forms of polyfunctional thiols (Vermeulen et al. 2006). Of these, 3-mercaptohexyl acetate (3MHA), the acetylated form of 3MH, has received considerable attention due to its positive contribution to flavour. Its distinctive flavour, often described as passion fruit-like, is sensorially apparent at exquisitely low levels in beer (4 ng/L). It was, like 3MH, first detected in passion fruit juice (Engel and Tressl 1991) and later found in a range of wines, particularly in Sauvignon blanc wines where it is an important component of the style’s flavour profile (Tominaga et al. 1996). There is also a growing appreciation of this flavour note in dry-hopped beers (Dennenlöhr et al. 2020). Studies on 3MHA perception in red wine have also shown that its presence can accentuate the perception of other polyfunctional thiols such as 4MMP (Rigou et al. 2014).

3MHA has not been detected in substrates prior to fermentation and is not believed to occur commonly in a bound form (though glutathionylated 3MHA has been detected in grape, Chenot et al. 2022a). Available evidence suggests that this compound is created through esterification of 3MH by yeast during fermentation (Fig. 1). Swiegers et al. (2006) demonstrated that this esterification was primarily due to the activity of alcohol acetyltransferase, in particular that coded for by the gene *ATF1* (Swiegers and Pretorius 2007). Other enzymes may also have a role to play, as evidenced by the fact that *ATF1* deletion did not completely prevent the esterification reaction from occurring.

Given the clear link between *ATF1* activity and 3MHA, an obvious strategy to boost levels of the acetyl ester would be to engineer yeasts for greater *ATF1* activity, possibly in combination with a reduced esterase activity to reduce the risk of the acetylated form reverting to 3MH downstream. The work of Swiegers et al. (2006) and Kiene et al. (2021) suggests that such an approach would be effective. Alternatively, one could screen yeasts for 3MHA production (Anfang et al. 2009), or screen yeasts that have a naturally high level of *ATF1* expression, e.g. those which are known to produce high levels of acetate esters such as 3-methylbutyl acetate. Acetyltransferase activity may be increased via adaptive evolution. This has been seen, for example, when saké yeast have been exposed to toxic levels of 1-farnesylpyridinium, a disruptor of acetyltransferase activity. Cell lines that developed resistance to these compounds showed acetyltransferase activities several-fold higher than in the parental train and consequently a higher production of acetate esters (Hirooka et al. 2005). A similar approach could be taken to encourage 3MH acetylation. Likewise, reduced esterase activity in saké yeasts through adaptive evolution has been demonstrated. In this case, activity of the *EST2* gene was disrupted via mutagenesis in an effort to maintain high levels of acetate ester (Fukuda et al. 1998), and a similar approach may be effective in preventing the loss of 3MHA.

Alternatively, one may take advantage of the esterification ability of non-*Saccharomyces* yeasts. Anfang et al. (2009), for example, demonstrated that a mixed culture of wine yeast and *Pichia kluyveri* was highly effective at promoting 3MHA levels. The synergistic effect observed may be due to liberation of 3MH by the wine yeast followed by esterification by the non-*Saccharomyces* partner (Anfang et al. 2009).

In addition to selecting or modifying yeast, one has the option of modifying the fermentation process to better support the formation and retention of 3MHA. Acetyltransferase activity in yeast is known, for example, to be influenced by oxygenation or the presence of unsaturated fatty acids in brewer’s wort, with both factors resulting in reduced *ATF1* gene expression and enzyme activity (Fujii et al. 1997). One would therefore expect greater 3MHA formation where excess oxygenation is avoided, or where a particularly clear (trub-free) wort is utilized at the beginning of fermentation. Likewise, an increase in temperature or wort gravity would be expected to promote *ATF1* expression and acetylation, as it does for acetate esters (Saerens et al. 2008).

3MHA levels may be determined to some extent by the type of conjugation of the precursor molecule. Winter et al. (2011) suggested that, despite the fact that 3MH is more readily released from cysteine-bound 3MH, higher levels of 3MHA were associated with higher level of the glutathione-bound precursor (Winter et al. 2011). While it is not yet clear why this would be the case, it is possible that the nitrogen fraction released through β-lyase activity in the cell may influence acetylation. Nitrogen concentration and composition are known to have an impact on acetyltransferase activity (Verstrepen et al. 2003). If it is indeed the case that the conjugation influences acetylation, this would suggest that those strategies designed to converted glutathionylated thiols to cysteinylated thiols (described above) may reduce the potential for 3MHA formation. Patel et al. (2010) noted that juice pasteurization resulted in wines with higher 3MHA levels. This occurred at the expense of 3MH, the levels of which were reduced by pasteurization (Patel et al. 2010). To what extent this might be influenced by the type of conjugation is not known. When considering 3MHA in fermented beverages, one must recognize that the molecule is highly volatile, and a gradual loss during fermentation or from the finished product is expected. As for other polyfunctional thiols, there is a risk of loss during active fermentation via CO_2_-stripping, and hopping early in fermentation is not advised for this reason. 3MHA is also highly susceptible to loss by hydrolysis. This instability was demonstrated by Makhotkina and Kilmartin (2012), who observed a drop in 3MHA levels in Sauvignon blanc wines over time, particularly at higher temperatures. The 3MHA was apparently converted to 3MH and acetic acid during the process (Makhotkina and Kilmartin 2012).

As is the case for other biotransformation reactions, considerably more is known about changes occurring during wine fermentation relative to brewery fermentations. It remains to be seen if our knowledge on thiol acetylation is transferrable to the brewing system. Also, despite the existence of multiple acetylated thiols in fermented beverages, to date most attention has been focused on 3MHA due to its low flavour threshold and positive flavour attributes. If other acetate forms of polyfunctional thiols also contribute in a significant way to flavour profiles of fermented beverages has yet to be established.

## Terpenes

Numerous essential oils are found in hops and form a complex combination of different volatile substances. The characteristic aroma of dry-hopped beers is mainly derived from these hop oils transferred to the beer during the brewing process, either in the brewhouse during late hopping in the whirlpool or in the cellar via different dry-hopping techniques. In this section, we will focus on the terpene alcohols, which are the main contributors to the fruity, citrus and floral aromas in final beers (Inui et al. 2013). This complexity arises from the high number of possible combinations of compounds, and the synergistic and masking effects among volatile and non-volatile beer constituents (Rettberg et al. 2018). The flavour thresholds of typical monoterpene alcohols are low. Some examples of monoterpene alcohols are shown in Table 1. Geraniol is described as having a lime, flower, hyacinth and rose aroma, and can be detected at a concentration of 6 µg/L, while β-citronellol has a citrus, floral, lime aroma and has a flavour threshold of 8 µg/L (Meilgaard 1982; Takoi et al. 2010a). Hop oils other than monoterpene alcohols can be found in higher concentrations in hops, but these compounds (e.g. myrcene, α-humulene, β-caryophyllene, β-farnesene) are less important for the aroma of both fresh and dried hops (Steinhaus and Schieberle 2000). While predicting the aroma impact of hops on the finished beer remains difficult, recent work has revealed the contribution of many individual oil components and effect of various process conditions.

**Fig. 2:**
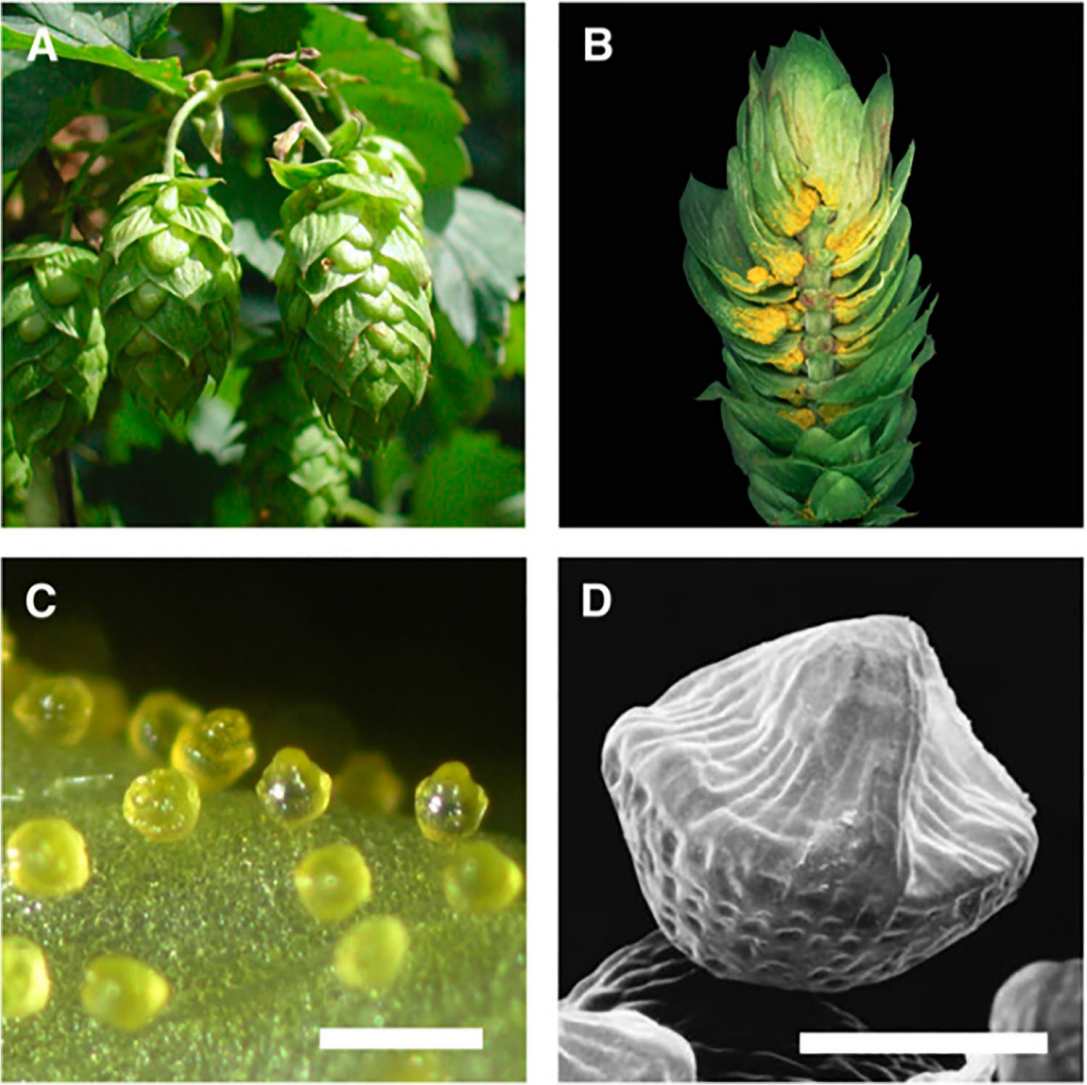
(**A**) Cones of the hop cultivar Taurus. Cones are ~ 5 cm in length. (**B**) Longitudinal section of a hop cone showing lupulin glands at the base of bracteoles. (**C**) A light microscopy image of ripe lupulin glands. Bar = 500 µm. (**D**) Scanning electron micrograph of a ripe lupulin gland showing the peaked appearance of the filled subcuticular sac. Bar = 100 µm (Nagel et al. 2008). Reprinted with permission

Hop oils are located mainly in glandular trichomes (lupulin glands), but also in leaves and flowers, as seen in Fig. 2. Myrcene is the most abundant hop oil > 80% v/w and is only available in the trichome, whereas linalool, α-humulene and β-caryophyllene are also available in leaves and flowers (Eri et al. 2000). Bitter acids, hop oils and prenyl flavonoids are all derived from pathways of terpene metabolism. Hop essential oils are synthesized from dimethylallylpyrophosphate (DMPP) and isopentenylpyrophosphate (IPP) to produce geranyl pyrophosphate (GPP), from which myrcene is synthesized through the action of terpene synthase (MTS) (Wang et al. 2008). Various species of hops convert many different terpenoids from the same substrate, and the subsequent addition of functional groups leads to different products (Tholl 2006; Nagegowda 2010).

The oil composition in hops is determined by genotype, with different hop varieties having different compositions (Oliveira et al. 1988; Saito et al. 1995). Not only does genetics determine the oil composition in hops but also the harvest time. Differences in the hop characteristics and chemistry of hops from different harvest maturities have been reported (Sharp et al. 2014; Matsui et al. 2016; Lafontaine et al. 2021). Studies have independently verified these results with Cascade and Willamette hops varieties in USA as well as with Saaz hop variety in the Czech Republic. Early harvesting resulted in lower essential oil composition from hops of the same variety, farm and year of harvest.

### Monoterpene alcohol glycosides

Terpenes and monoterpene alcohols are found in hops in free form as well as bound to other molecules. Usually, they consist of an aglycone and a glycone, the latter being an activated sugar, and the former, a non-sugar moiety, such as linalool or geraniol. Monoterpene alcohol glycosides were first identified in grapes but are also found in other fruits such as apples. Plants use glycosides as a way to store certain molecules that are important for metabolism (Wills and Scriven 1979; Williams et al. 1982). It was observed in certain plants, that as flowers begin to open, there is a higher amount of flavour substances in the bound form than in the free form (Watanabe et al. 1993). Glycosylated aroma compounds are more water-soluble and less reactive, facilitating their transport around, and storage within, plant tissues (Winterhalter and Skouroumounis 1997). Glycosides are synthesized by a glucosyltransferase enzyme, which adds an activated sugar to an aglycone. Rhamnose, galactose, xylose and glucuronic acids have been identified as activated sugars (Bowles and Lim 2010). Bowles and Lim in 2010 highlighted the importance of glycosyltransferases. These enzymes transfer sugars to different acceptors, including aroma components, such as terpenes. These glycosides, now more soluble, due to their increased hydrophilic nature, than the free aglycones, can now be easily transported from the cytosol (Bowles and Lim 2010). Glycosylation also stabilizes compounds, allowing the plant to store components, such as aroma compounds, which can be used later as repellents or attractants as needed (Bouwmeester 2006).

In hops, monoterpene alcohols can be found in a glycosidically bound form, and their levels, as well as the levels of terpenes and terpene alcohols, are dependent on the hop variety (genetic factors) (Takoi et al. 2016; Lafontaine et al. 2021). The first report of glycosides and their corresponding volatile aglycones in hops was done by Goldstein et al. in 1999 and quantified by Kollmannsberger et al. 2006; the aglycones detected included linalool, β-citronellol and phenylethanol (Goldstein et al. 1999; Kollmannsberger et al. 2006). To understand the potential of these bound aroma components, the formation of hop essential oils in the plant and during ripening is key to choosing the right hop variety and time of harvest. This way, the hop aroma potential of these hops can be more fully exploited. Haslbeck et al. reported a higher concentration of glycosides in fertilized hops relative to unfertilized hops. Wilhelm (2013) measured the amount of glycosides present in four hop varieties indirectly by determining the aglycone released enzymatically by Rapidase F64 enzyme.

### Monoterpene alcohol release through β-glucosidase activity

In the last decade, research has also revealed how yeast can release and enhance characteristic hop aromas to the beer that were ‘hidden’ in the plant. These aromas can be liberated only through enzymatic activity, such as that of yeast during fermentation (Fig. 1). Yeast therefore has the ability to enhance the hop aroma characteristic of dry-hopped beer. Promotion of such biotransformation reactions in brewing may obviate the need for additional hops, or specialist hops, to achieve particular hop aroma profiles in beers (Goldstein et al. 1993; Kollmannsberger et al. 2006). The inter sugar bond present in glycosides can be cleaved by a 1,4-β-glucosidase (EC.3.2.1.21), first reported by Gunata et al. (1988) and Sarry and Günata (2004). While hops contain glycosidically bound monoterpene alcohols that can be enzymatically cleaved, it is under debate whether their release has any perceivable impact on beer aroma.

Sharp et. al., after screening yeasts for their β-glucosidase activity, and performing fermentations with selected yeasts in wort dry hopped with three different hop cultivars, found no significant difference in the terpene alcohol concentrations. This was probably due to an inhibition in the expression of the enzyme by the presence of glucose, and/or the anaerobic conditions (Sharp et al. 2017a). Serra Colomer and colleagues had similar results when screening for β-glucosidase activity in *Brettanomyces* yeasts and found no correlation between the strains with high enzyme activity and the geraniol content in the beers after dry-hopping (Serra Colomer et al. 2020).

Van den Bremt used the term bio-generation to describe production of novel flavours in beer, by using the yeast *Candida methanolovescens*, the β-glucosidase enzyme of which produced salicyl aldehyde from salycin, which has a characteristic almond aroma (Van Den Bremt et al. 2001). In 2003, Vanderhagen introduced the concept of beer bio-flavouring using different yeast strains, either to reduce aldehyde content or to release aromatic substances in hops (Vanderhaegen et al. 2003). In *S. cerevisiae*, the enzyme exo-β-glucanase (encoded by the *EXG1* gene) was found to cleave the inter-sugar bond in glycosides and to be released independently of the carbon source. Olivero et al. reported in 1985 that the overproduction of this enzyme resulted in an increase in volatile compounds (Olivero et al. 1985; Gil et al. 2005). Daenen et al. in 2008 looked for this enzyme activity in 59 yeasts, including 9 lager yeast strains, 27 ale yeasts strains, one yeast strain with deleted *EXG1* gene/YLR300delta and 18 *Brettanomyces* spp. yeasts. Only two yeast strains exhibited β-glucanase activity, a brewing *S. cerevisiae* strain and a *Brettanomyces custersii* strain isolated from a lambic beer fermentation, the latter with 10 times more β-glucosidase activity than the first one, and even higher activity in co-culture with *S. cerevisiae* (Daenen et al. 2008)*.* In 2017, Sharp et al. conducted extensive research on β-glucosidase activity of 80 different yeast strains including commercial *S. cerevisiae* strains for brewing, wine, saké and baking, as well as *Brettanomyces anomala*, *Brettanomyces bruxellensis*, *Candida versatilis*, *Kluyveromyces marxianus*, *Scheffersomyces stipitis*, *Saccharomyces pastorianus and Debaryomyces nepalensis.* The β-glucosidase activity did not depend on the species, though highest activity was observed for two *Brettanomyces* strains. No clear benefits were seen when hopping regime was modified (Sharp et al. 2017b). Serra Colomer, inspired by the studies of Daenen et al. (2008) and Sharp et al. (2017a, b), continued yeast screening for β-glucosidase activity, this time focusing only on *Brettanomyces spp.* strains. The results showed that *Brettanomyces anomalus* and *Brettanomyces bruxellensis* exhibited the highest activity. And, because it has been reported that two open reading frames, which encode two β-glucosidase enzymes, are present in *Brettanomyces spp.*, the study also focused on strains with and without these ORFs. In this study, there appeared to be no direct correlation between the β-glucosidase activity and hop aroma in the beers treated with *Brettanomyces* for primary or secondary fermentation (Serra Colomer et al. 2020).

Gunata et al. performed trials with an immobilized β-glucosidase enzyme isolated from *Candida molischiana* 35M5N and successfully released after 7 h the flavour compounds linalool and geraniol from wine and fruit juices (Gunata et al. 1988). In 2016, Vervoort et al. screened 428 *Brettanomyces* yeasts for β-glucosidase activity and isolated enzymes from these strains and compared them to β-glucosidases from *Aspergillus niger* and *Prunus dulcis* (almond). *B. anomalus* presented the highest activity (Vervoort et al. 2016).

Some commercial enzymes are already available in the market; these include Aromazyme, commercialized by Lallemand and released in 2020. This has been tested in some commercial breweries and has shown apparently promising results—releasing hop aroma volatiles. Other enzymes, such as Rapidase or Sumyzime, have been used to release monoterpene alcohols that were bound to sugar moieties (Takoi et al. 2017). The results by Lafontaine et al. open the door to new possibilities for using other enzymes besides β-glucosidase such as α-L-arabinofuranosidase and/or α-L-rhamnosidase to release these bound compounds (Lafontaine et al. 2021).

### Terpene alcohol conversion

The importance of citrus, fruity and floral aromas in beer has transcended the craft beer scene to the non-alcoholic beer (NAB) trend; Rettberg et. al. reported earlier this year that enhanced hoppy aromas are preferred in NAB (Rettberg et al. 2022). The positive synergy between geraniol, linalool and β-citronellol in beers was also reported by Takoi and his collegaues (Takoi et al. 2010a). The β-citronellol content in beer cannot be increased merely by adding hops, and it appears that yeast biotransformation is critical in determining levels of this compound. Already in 1986, it was reported that yeast can transform enzymatically certain terpenoids into other terpenoids, including the reduction of geraniol to β-citronellol and the hydrolysis of geranyl acetate and isobutyrate to geraniol (Fig. 1) (Lam et al. 1986). Since then, conversions of various mono- and sesquiterpenes have been demonstrated (King and Dickinson 2000; Takoi et al. 2010b; Praet et al. 2012). During the fermentation of dry-hopped beer, Takoi et al. observed a slight decrease in linalool and α-terpineol, followed by an increase in β-citronellol and nerol, and then an increase of geraniol (Takoi et al. 2010b). Already in 2000, King et al. suggested a bioconversion of geraniol into linalool and linalool into α-terpineol (King and Dickinson 2000). These compounds can be further metabolized (e.g. via esterification/hydrolysis) (Lam et al. 1986; King and Dickinson 2000; Chatterjee and Bhattacharyya 2001). To enhance the citrus, fruity, floral aroma in beers through increasing geraniol and β-citronellol content in the final beer, Reyes et al. from Sierra Nevada Brewing Co. performed dry-hopping trials with Cascade hops and added the hops in different stages of fermentation. Higher concentrations of geraniol, linalool, nerol and some ethyl esters were found in these beers, and suggested that the hop dosing time with the presence or absence of yeast influences the flavour profile in beer (Moutsoglou et al. 2018; Reyes 2019).

Looking into the transformation of geraniol into other monoterpene alcohols, such as β-citronellol, Takoi noted that the yeast growth phase has an influence on the transformation of geraniol (Takoi et al. 2010a). Geraniol is converted mainly 2–4 days after initiation of fermentation, and it appears that the enzyme NADPH dehydrogenase 2 (encoded by *OYE2*) is responsible for the reduction of this monoterpene alcohol to β-citronellol (King and Dickinson 2000; Yuan et al. 2011). Later, in 2013, it was reported that overexpression of *OYE2* increased the reduction to 87% in comparison to 50% with a control strain (Steyer et al. 2013). Further confirmation came from Zhao and colleagues, who reported that the deletion of *OYE2* or *ATF1* genes led to an improved geraniol production by 1.7-fold or 1.6-fold in batch fermentation (Zhao et al. 2017). The activity of this enzyme has been observed in the cytoplasm, mitochondrion and nucleus of the yeast (Holt et al. 2018).

Serra Colomer et al. in 2020, while screening for β-glucosidase activity in *Brettanomyces spp*. species, found that the strains with lowest β-glucosidase activity showed the highest concentrations in β-citronellol (up to 31.5 µg/L). It was also suggested in that study that the reduction from geraniol to β-citronellol could be directly influenced by the oxidoreductase proteins BbHye2 and BbHye3 (Serra Colomer et al. 2020). Similar results to the ones from Reyes et al. in 2019, on hop timing and effect on biotransformation, were reported by Williams (2019), as a mid-fermentation dry hop addition increased the β-citronellol concentration in the final beers (Williams 2019).

Terpene contents in fermented beverages are not only influenced by reduction and isomerization reactions but also by changes in acetylation that are likewise mediated by fermentative yeasts (King and Dickinson 2003). As for the thiol ester 3MHA described above, the yeast creates acetylated forms of monoterpene alcohols via acetyltransferase activity, with the enzyme produced by the *ATF1* playing a significant role (King and Dickinson 2000; Steyer et al. 2013; Zhao et al. 2017). Examples include geranyl- and citronellyl-acetates (King and Dickinson 2003). It is also believed that the ester forms, e.g. geranyl isobutyrate or geranyl acetate, may be hydrolysed back to the geraniol form via acetate esterase activity of the yeasts (Peacock et al. 1981). Levels of monoterpene alcohols in beer may therefore be the net result of these competing reactions. These changes may be significant as the form of the compound may influence the flavour threshold level or the flavour attribute. Geranyl acetate has been reported to have a lower flavour threshold than geraniol, and also a more lavender character (Pardo et al. 2015).

It can be noted here that monoterpene alcohols can exist in acetylated and non-acetylated forms in the raw material. Hops, for example, contain both geranyl acetate and geraniol, with hop varieties varying greatly in their relative contents of both (Forster et al. 2014). The work of Forster and co-workers (2014) showed how hop varieties, e.g. Cascade, Hallertau Blanc and Polaris, all containing high concentrations of geranyl acetate, typically produced beers with a low level of geranyl acetate but a high level of geraniol. This suggested that hydrolysis of the compounds occurred during fermentation, as suggested previously by Lam et al. (1986).

The potential impact of non-conventional yeast on terpene alcohol ester levels is not clear. At least one study, involving *Williopsis saturnus*, showed the production of geranyl acetate from a hopped wort. This compound was not observed in the reference ale yeast (Liu and Quek 2016). Similarly, a higher concentration of geranyl acetate was observed in wine co-fermented with *L. thermotolerans* (Korenika et al. 2020). It may be assumed that yeast choice has an influence on the relative concentration of terpene alcohols and esters; further studies are however required to prove this assumption.

Hop terpenoids undergo significant chemical and functional modification during the brewing process (oxidation, hydrolysis and isomerization). For example, the amount of hop terpenoids decreases with increasing boiling time (Kishimoto et al. 2005), and a later addition is recommended in order to achieve a higher hop oil content in the beer, i.e. addition in the whirlpool or dry hop additions are more effective than traditional wort boil addition. Takoi et al. (2016) demonstrated the benefit of later dry hopping to boost the concentrations of geraniol. Later hopping appeared to avoid the consumption of geraniol, either derived directly the hop or via hydrolysis of an ester form, during active yeast growth (Takoi et al. 2016). It has been reported that oxygenated terpenoids, as they have a higher solubility, remain in higher concentration in the final beer (Sharp et al. 2017a). Apart from the above-mentioned biotransformation reactions, during fermentation, the terpene hydrocarbons from hops are lost due to their hydrophobic nature; either the yeast cell wall components adsorb them, or they migrate to the foam layer (Lam et al. 1986; King and Dickinson 2003; Praet et al. 2012).

### Alternative ways to increase terpene alcohols

Monoterpene alcohol concentrations in beer can also be increased through de novo formation by yeast. Genetic engineering of brewer’s yeast for monoterpene alcohol production has been demonstrated, but these are not widely used yet because of legislation and lack of consumer acceptance. Carrau et al. (2005) demonstrated that low levels of geraniol and linalool are naturally synthesized de novo by strains of *Hanseniaspora uvarum* and *S. cerevisiae* through the isoprenoid pathway. By introducing a linalool synthase encoding gene from the plant *Clarkia breweri,* increased linalool yields could be achieved (Herrero et al. 2008). Similarly, heterologous expression of a geraniol synthase from basil in *S. cerevisiae*, together with mutagenesis of the native *ERG20* gene, resulted in geraniol yields over 5 mg/L (Fischer et al. 2011). More recently, an industrial brewing yeast was engineered to produce both linalool and geraniol in similar ratios and amounts as those found in dry-hopped beers (Denby et al. 2018). Here, geraniol and linalool synthesis were accomplished by heterologous expression of a geraniol and linalool synthase from basil and mint, respectively. Monoterpene alcohol levels were controlled by modulating expression of the heterologous enzymes and two downstream enzymes in the isoprenoid pathway.

## Conclusions and future outlook

The continued and growing interest in hop aromas in beers has focused attention on those factors in the process that can be modified to maximize aroma or steer hop aroma in particular directions. Fortunately, brewers have a number of tools at their disposal. Levels of hop oils and flavour compounds, as well as their composition in beer can be adjusted, not only by changing the amount, type or timing of hop addition, but also by promoting specific biotransformation reactions mediated by yeasts during the fermentation process. Biotransformation of hop compounds during brewing is a relatively new field of study; investigations in recent years have highlighted, not only the importance of these yeast-mediated reactions, but also our lack of fundamental knowledge regarding the genetic and biochemical processes responsible. Further investigations are expected to facilitate the effective exploitation of biotransformation reactions in brewing and other beverage fermentation processes.

In regard to β-lyase activity, challenges still remain in increasing the overall conversion rate of precursors to free thiols and better understanding the role of yeast enzymes other than Irc7p. Research in this field has also been limited by analytic capabilities. Polyfunctional thiol concentrations, for example, are present in the ng/L range and therefore require sensitive methods (Capone et al. 2015; Dennenlöhr et al. 2020), while methods for accurately measuring concentrations of cysteinylated and glutathionylated precursors directly from the raw materials have only recently been developed (Roland et al. 2016; Chenot et al. 2022a).

The release of monoterpene alcohols from glycoside precursors has been studied by means of screening the β-glucosidase enzyme activity from different microorganisms. The quantification and classification of monoterpene alcohol glycosides in hops have been challenging, and different authors report a wide range of concentrations of these compounds. It has also been speculated that other enzymes more specific to the sugar moieties in the glycosides could be used to release these aroma components, not only β-glucosidases but also α-L-arabinofuranosidases, α-L-rhamnosidases and terpene-isomerases could also be used (Lafontaine et al. 2021). And synergistic interactions between different monoterpene alcohols and volatile thiols play an important role in the hop aroma flavour of beer even at concentrations as low as 5 µg/L of geraniol (Takoi et al. 2016). Functional and genetic characterization of the remaining terpenoid synthases, regulation and transformation by yeast is still required.

A greater understanding of biotransformation reactions and how they can be usefully manipulated is important, not only for production of beers with a desirable aroma profile, but also to ensure the sustainability of brewing. The dry-hopping process is particularly costly with respect to water and raw material use (considerable loss of beer occurs when hops are removed from the system) (Hauser et al. 2019). Any intervention that increases the transfer of aroma compounds, thereby limiting the demand for hop raw material, will have a direct impact on process efficiency, while simultaneously reducing reliance on a crop that is highly susceptible to damage by climate change (Kind and Kaiser 2020). Also, spent hops and industrial side streams arising from creation of hop products contain high levels of bound aroma compounds and therefore have the potential to be re-utilized via yeast biotransformation reactions, or through the use of exogenous enzymes. Biotransformation reactions, in addition to changing the quantities of hops required in the process, can also serve to change the aromatic qualities of the hops used. The most sought-after hop varieties, those, e.g. with high levels of tropical fruit flavour notes are costly and grown only in selected areas. Biotransformation reactions have the potential to liberate exotic fruit notes from even standard hop varieties, thereby increasing the range of flavours that can be produced from local, or traditional, hop varieties.

The growing popularity of beers with hoppy aroma coincides with a growing interest in beers with low, or no, alcohol (Conway 2020). Methods used to produce these beers have the unfortunate side-effect of removing most of the volatile aroma compounds that are generated by yeast during fermentation. An increase in hop-derived aroma compounds from biotransformation reactions may serve to compensate for the loss of these fruity and floral aromas, thereby producing more palatable low-alcohol beers (Brendel et al. 2020; Lafontaine et al. 2020). Also, as highlighted in this review, *Saccharomyces* yeasts are not the only yeast species capable of increasing hop aroma compounds during fermentation. It is clear that much of the aromatic potential of hops could be tapped by exploiting the vast genetic diversity of available yeast species. In particular, it will be of value to test the biotransformation potential of those yeasts that are already being considered for low-alcohol beer brewing due to their maltose negativity (Gibson et al. 2017).

Furthermore, industrial trials considering the costs of these special process parameters should be performed. Continuous analysis of the impact of climate change on hop oil composition (low water and high-temperature stress), which will result in breeding new strains should be performed (Eriksen et al. 2021).

Yeasts have long been known to contribute greatly to beer quality, not only producing alcohol and CO_2_, but also determining beer character through the production of various flavour-active metabolites and the removal of wort aldehydes, as well as stabilization of beers through the production of sulphite and removal of oxygen. To add to this, there is now a growing appreciation of the role that yeasts play in manipulating hop aroma. It is expected that as our understanding of this property improves, so will our ability to develop products with tailored hop aroma profiles in an efficient and sustainable way.
